# Community integration enhances migrants’ satisfaction with primary care across districts with varying economic levels: survey evidence from Guangzhou, China

**DOI:** 10.3389/fpubh.2025.1604736

**Published:** 2025-06-11

**Authors:** Yao Xiao, Sisi Zhong, Lingshan Chen, Diwen Xiao, Wenhao Huang, Shangjian Wu, Luwen Zhang

**Affiliations:** ^1^School of Health Management, Guangzhou Medical University, Guangzhou, China; ^2^School of Medicine and Health Management, Tongji Medical College, Huazhong University of Science and Technology, Wuhan, China; ^3^School of Health Management, Southern Medical University, Guangzhou, China; ^4^Center for Health Policy and Governance of Guangdong Provincial Social Science Research Base, Southern Medical University, Guangzhou, China; ^5^Institute of Public Administration, Party School of the Guangdong Provincial Committee of CPC, Guangzhou, China; ^6^School of Pharmaceutical Sciences, Guangzhou Medical University, Guangzhou, China

**Keywords:** migrant population, primary care, satisfaction, social integration, economic level

## Abstract

**Background:**

This study investigated the satisfaction of migrant populations in Guangzhou with primary care and explored how community integration and economic development at the district level influenced this satisfaction. This study aimed to provide empirical evidence and recommendations for improving primary care utilization and community integration among migrant populations.

**Methods:**

We conducted a stratified random sampling of 1,996 migrant individuals from seven districts in Guangzhou. A custom-designed questionnaire was used to collect data on demographic characteristics, satisfaction with primary care, and community integration, including willingness to seek help, neighborhood interactions, and participation in community activities. Multi-variate linear regression analysis was used to identify factors associated with satisfaction with primary care.

**Results:**

The mean satisfaction score for primary care was 3.29 ± 0.76. Participation in community activities was positively associated with satisfaction, with participants often reporting higher scores (increases of 0.08 and 0.28 points, respectively; *p* < 0.05). Greater neighborhood interaction and willingness to seek assistance from local authorities also increased satisfaction (0.11 and 0.37 points, respectively; *p* < 0.05). Residing in districts with moderate or good economic conditions further enhanced satisfaction (0.37 and 0.10 points, respectively; *p* < 0.05).

**Conclusion:**

Although migrant populations in Guangzhou generally report high satisfaction with primary care, their level of community integration remains limited. Enhancing community public services and fostering a stronger sense of community belonging are promising strategies for improving primary care management and services for migrant populations.

## 1 Introduction

Industrialization and urbanization in China have driven a massive migration of residents to cities. By the end of 2022, migrant populations accounted for approximately 18% of the total population, according to the China Migrant Population Development Report (2022). These individuals face heightened health risks owing to occupational hazards and poor living conditions. In their destination cities, migrant populations often encounter inadequate access to immunization, infectious disease control, and occupational health protection (1). Moreover, they frequently face disadvantages when accessing routine medical services and insurance benefits (2). The World Health Organization (WHO) emphasizes that everyone should have access to essential healthcare (3). As such, ensuring that the primary care needs of migrant populations are met is a critical public issue that must be addressed (4). In China, primary care is primarily provided by primary care institutions, including township hospitals and community health centers within community settings.

The community not only serves as the primary location for migrant populations to access primary care services, but also as their foothold and foundation for integration into urban life. Community integration refers to the process of interaction, exchange, adaptation, and acceptance among individuals, groups, and cultures, and reflects the degree of participation, identification, and acceptance within a specific society. Most studies have indicated that community integration facilitates the utilization of primary care services by migrant populations. For instance, research by Bu et al. found that older adult migrant populations with better community integration tend to use more public health services (5). Jing discovered that enhancing the community integration of migrant parents effectively increased the utilization rate of healthcare services among migrant children (6). Studies have also suggested that receiving public health services can improve community integration among migrant populations (7). These findings highlight a mutually reinforcing relationship between community integration and health service utilization. Strengthening community integration can enhance the access of migrant populations to primary care services, reinforcing their sense of identity and community integration. However, academic research on the relationship between the access of migrant populations to primary care services and community integration remains limited and warrants further attention.

In urban China, primary care provision is predominantly funded at the district level by government fiscal income. Therefore, the economic development level of districts significantly influences the primary medical service capacity and institutional construction level of the entire region (8). In economically developed districts, governments have stronger financial capabilities to support the development of primary care, enabling them to create high-quality medical environments, purchase advanced medical equipment, attract a large number of medical professionals, and provide higher-quality primary medical services (9). In contrast, economically underdeveloped areas face relatively backward primary medical service capabilities and institutional construction levels due to limited financial resources (10). These regional disparities in primary medical service capabilities and institutional construction levels further affect the satisfaction of migrant populations with primary care (11).

However, the satisfaction of migrant populations with primary care is not only influenced by primary care provision, but also impacted by their perception and experience of integrating into urban life (12). Some other studies suggest that migrants working or living in higher economic development regions may face more difficulty in integrating into local community, which may negatively affect their satisfaction with primary care (13). This duality underscores the need for context-specific policy interventions to reconcile economic growth, community integration promotion and primary care provision. Therefore, alongside enhancing primary healthcare capacity and institutional development, attention must be given to migrants’ perceptions and experiences of urban integration. Systematic approaches involving cross-sector collaboration and policy coordination are required to address the complex interactions among economic development, social integration, and healthcare provision, thereby improving migrants’ overall satisfaction with primary care (14).

Guangzhou, as the capital of Guangdong Province and a megacity of southern China, is a major destination for migrants with a resident population exceeding 22 million and managing over 10 million migrant residents (15). Addressing the healthcare needs of the migrant population and ensuring their health and sustainable development are the primary responsibilities of the Guangzhou government (16). To ensure migrant populations access basic medical resources and promote social integration, Guangdong Province has enacted policies guaranteeing migrants’ equal access to primary care, family planning, public cultural and sports services, employment services, and compulsory education (17). The government also prioritizes migrant health education and promotion by establishing model enterprises, schools, and healthy families, using diverse approaches to encourage migrants’ local integration, health management participation, and service utilization (18).

Therefore, this study investigated the satisfaction of migrant populations in Guangzhou with primary care and analyzed the association between community integration and district-level economic development with satisfaction. It also explored the factors that enhance the utilization of primary care among migrant populations.

## 2 Methods

### 2.1 Sample size

Guangzhou comprises 11 districts, with its 7 central urban districts (Tianhe, Yuexiu, Haizhu, Liwan, Baiyun, Huangpu, and Panyu) collectively hosting 83.98% of the city’s migrant population (19). Therefore, these 7 urban districts were selected as the study sites.(See Appendix 1 for details).

The minimum sample size was calculated using a standard formula for the population proportion estimation (20):


$$n=\frac{z^{2}\hat{p}(1-\hat{p})}{\Delta^{2}}$$

where *n* is the sample size, *z* is the confidence interval, is the estimated population proportion, and *Δ* is the margin of error.

Based on the 2019 national migrant population proportion of 17%, with a margin of error of 2% and a 95% confidence interval, the minimum sample size was calculated as 1,355. Considering a 10% nonresponse rate, the final minimum sample size was adjusted to 1,491.

**Figure 1:**
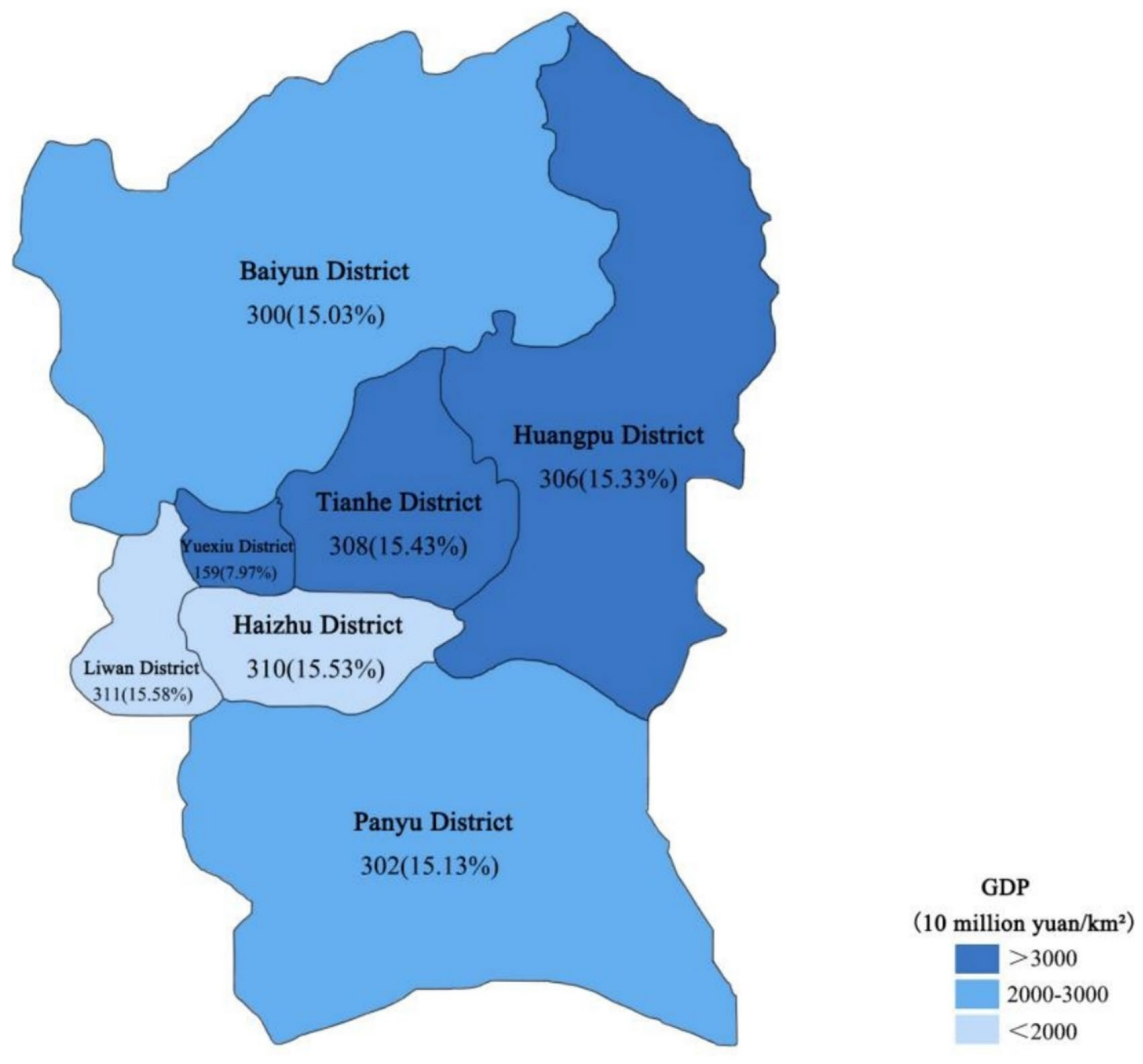
Regional economic characteristics and sampling methodology of the investigated areas in Guangzhou.

We categorized the 7 sampled districts into three economic levels (low, medium, and high) based on their 2019 GDP levels and ensured balanced sampling across these levels. A total of 2,050 questionnaires were distributed. After excluding invalid responses, we obtained 1,996 valid questionnaires with a response rate of 97.37%. The distribution was balanced across the economic levels: high (773, 38.73%), medium (602, 30.16%), and low (621, 31.11%). The specific sampling data for each district are shown in Figure 1.

### 2.2 Questionnaire and survey

The questionnaire was designed based on a literature review, expert interviews, and a pilot survey. Trained investigators conducted the survey using a paper-based questionnaire. The questionnaire primarily covered demographic information (e.g., age, sex, household registration, occupation, length of residence), satisfaction with primary care, and community integration.

The survey was conducted between January and March, 2019. Paper questionnaires were distributed through community-based outreach and recruitment at community health centers, migrant service centers, labor markets, and other channels within the sub-districts (towns). Questionnaires were checked for quality immediately upon completion. The participants provided informed consent and cooperation to ensure the integrity and consistency of their responses. Face-to-face interviews were conducted to ensure complete and consistent data collection. Participants met the following criteria: (1) residing in Guangzhou for at least 6 months; (2) non-Guangzhou household registration; and (3) able to fill out the questionnaire independently or with assistance; (4) age ≥18 years old. All participants were informed and agreed to participate in the study.

### 2.3 Main variables

#### 2.3.1 Satisfaction with primary care

Satisfaction with primary care was defined as the migrants’ comprehensive evaluation of community-based healthcare services across four domains: service quality, facility environment, staff attitudes, and procedural efficiency. Responses were quantified using a 5-point Likert Scale (1 = Very Dissatisfied, 5 = Very Satisfied).

#### 2.3.2 Community integration

With rapid economic growth and internal migration, promoting community integration of the migrant population has become a key management focus. Communities are both the primary living spaces for migrants and central sites for governance and public service delivery. As a subset of social integration, community integration emphasizes interactions between residents and their communities and their outcomes. Primary care, as an integrated public service model, plays a crucial role in advancing migrant social integration (21). Based on China’s context, scholars have developed a four-dimensional framework-economic integration, social adaptation, cultural acquisition, and psychological identification that includes help-seeking, neighborhood interaction, and community participation (22). *The 2013 Survey on Social Integration of the Migrant Population in China* confirmed the validity of indicators like community participation and help-seeking willingness (23).

In this study, community integration was measured through three items:

Neighborhood interaction: it refers to the social interaction and emotional relationships among nearby residents, including various activities such as visiting others, providing mutual support, and offering mutual care (24). Neighborhood relationships are a key factor in the social integration of migrant populations because they can provide employment opportunities, build social trust, and offer social activities (25). Neighborhood networks are considered a fundamental component of community integration, particularly for vulnerable groups in society (26).

Participation in community activity: this reflects an individual’s concern for and willingness to participate in or engage in community affairs. Migrants’ involvement in public activities helps foster a sense of belonging to the city (27).

Willingness to seek help: this indicates the degree of willingness to seek help from local community service offices or neighborhood committees when facing difficulties. This reflects an individual’s tendency to seek help when problems arise and indicates the level of community integration. If migrant populations are more inclined to make friends with locals or seek help from employers, the government, or lawyers rather than remaining within circles of fellow migrants, their social integration is generally higher (28).

#### 2.3.3 Covariates

The covariates included age, sex, household registration, marital status, educational level, occupation, length of residence, average monthly income, and industry classification. Occupations were categorized according to the Three-Industry Classification Regulations (2012) in China into service industry (e.g., service staff, salespersons, and cashiers), manufacturing industry (e.g., construction workers), and others (occupations not easily categorized).

#### 2.3.4 Statistical analysis

Valid questionnaires were entered into Epidata 3.1 to build the dataset, and statistical analysis was performed using Stata MP 16.0. Descriptive statistics included means and percentages. Chi-square and rank-sum tests were conducted for the relevant categories. Stepwise multi-variate linear regression was used to analyze the factors influencing migrant populations’ satisfaction with primary care. Differences were considered statistically significant at *p* < 0.05.

## 3 Results

### 3.1 Characteristics of respondents

Table 1 presents the sociodemographic characteristics of the migrant population. Among the 1,996 migrant individuals surveyed in Guangzhou, 1,041 were female (52.15%), and the majority were young adults who were aged 18–29 years (53.06%). Regarding educational background, a high proportion held junior high school or high school diplomas (54.01%). Married people accounted for 51.75% of the population. Most migrant individuals held rural Hukou registrations (62.88%), and the majority resided locally for 1–5 years (51.75%). The average satisfaction score for primary care was 3.29 ± 0.76.

**Table 1:** Characteristics of participants and disparity analysis across levels of GDP (*N* = 1996).

Variables	Category	All *n* (%)	Levels of GDP *n* (%)		
			Low	Medium	High
Total		1996 (100.0)	621 (31.11)	602 (30.16)	773 (38.73)
Sex	Male	955 (47.85)	259 (41.71)	330 (54.82)	366 (47.35)
	Female	1,041 (52.15)	362 (58.29)	272 (45.18)	407 (52.65)
Age	≤29	1,059 (53.06)	350 (56.36)	330 (54.82)	379 (49.02)
	30–39	510 (25.55)	153 (24.64)	160 (26.58)	197 (25.49)
	≥40	427 (21.39)	118 (19.00)	112 (18.60)	197 (25.49)
Education	Primary and lower	90 (4.51)	19 (3.06)	19 (3.16)	52 (6.73)
	Junior/senior high	1,078 (54.01)	360 (57.97)	347 (57.64)	371 (47.99)
	College and above	828 (41.48)	242 (38.97)	236 (39.20)	350 (45.28)
Medical insurance	Yes	1,484 (74.35)	474 (76.33)	483 (80.23)	527 (68.18)
	No	512 (25.65)	147 (23.67)	119 (19.77)	246 (31.82)
Marriage	Single/divorced	963 (48.25)	313 (50.40)	302 (50.17)	348 (45.02)
	Married	1,033 (51.75)	308 (49.60)	300 (49.83)	425 (54.98)
Hukou registration	Rural	1,255 (62.88)	427 (68.76)	291 (48.34)	537 (69.47)
	Urban	741 (37.12)	194 (31.24)	311 (51.66)	236 (30.53)
Monthly income (RMB)	≤3,500	560 (28.06)	152 (24.48)	167 (27.74)	241 (31.18)
	3,501–6,500	1,159 (58.07)	402 (64.73)	279 (46.35)	478 (61.84)
	≥6,501	277 (13.88)	67 (10.79)	156 (25.91)	54 (6.99)
Years of migration	≤1 year	479 (24.00)	174 (28.02)	123 (20.43)	182 (23.54)
	1–5 years	1,033 (51.75)	267 (43.00)	340 (56.48)	426 (55.11)
	≥ 5 years	484 (24.25)	180 (28.99)	139 (23.09)	165 (21.35)
Occupation	Service Industry	1,231 (61.67)	457 (73.59)	373 (61.96)	401 (51.88)
	Manufacturing Industry	312 (15.63)	72 (11.59)	131 (21.76)	109 (14.10)
	Others	453 (22.70)	92 (14.81)	98 (16.28)	263 (34.02)
Satisfaction to primary care (mean ± SD)		3.29 ± 0.76	3.13 ± 0.87	3.53 ± 0.80	3.23 ± 0.58

### 3.2 Community integration of the migrant population

Overall, more than half the respondents reported a relatively negative social integration (Table 2). The frequency of neighborhood interactions was low, with 62.88% reporting infrequent interactions and 18.93% reporting no interactions. Regarding participation in community activities, the majority either did not participate (36.97%) or participated infrequently (50.50%). In terms of willingness to seek help from street offices or residents’ committees when faced with difficulties, 43.14% reported low willingness and 35.97% expressed no willingness. Notably, more economically developed regions exhibited poorer overall social integration, with statistically significant differences (*p* < 0.001).

**Table 2:** Disparities in community integration across levels of GDP.

Variables	Category	Total *n*(%)	Levels of GDP *n* (%)			Chi	*p*
			Low	Medium	High		
Neighborhood interactions	Rarely	378 (18.93)	118 (19.00)	67 (11.13)	193 (24.97)	127.52	<0.001
	Sometimes	1,255 (62.88)	346 (55.72)	384 (63.79)	525 (67.92)		
	Often	363 (18.19)	157 (25.28)	151 (25.08)	55 (7.11)		
Community activity participation	Rarely	738 (36.97)	197 (31.72)	214 (35.55)	327 (42.30)	27.16	<0.001
	Sometimes	1,008 (50.50)	359 (57.81)	296 (49.17)	353 (45.67)		
	Often	250 (12.53)	65 (10.47)	92 (15.28)	93 (12.03)		
Willingness to seek help	None	417 (20.89)	141 (22.70)	154 (25.58)	122 (15.78)	21.84	<0.001
	Low	861 (43.14)	267 (43.00)	244 (40.53)	350 (45.28)		
	High	718 (35.97)	213 (34.30)	204 (33.89)	301 (38.94)		

### 3.3 Satisfaction with primary care

The satisfaction scores for primary care services varied significantly across different levels of neighborhood interactions, community activity participation, and willingness to seek help, with all differences being statistically significant (*p* < 0.001) (Table 3). Those who often participated in neighborhood interactions or community activities generally reported higher satisfaction scores, especially in medium-GDP regions. However, economic development level was inversely related to social integration, with more developed regions showing poorer integration and lower satisfaction scores in some categories.

**Table 3:** Satisfaction with primary care across levels of community integration and GDP per capita.

Variables	Category	Levels of GDP			*F*	*p*
		Low	Medium	High		
Neighborhood interactions	Rarely	3.20 (1.20)	3.72 (0.74)	3.21 (0.65)	17.02	<0.001
	Sometimes	3.09 (0.80)	3.40 (0.75)	3.23 (0.55)		
	Often	3.17 (0.71)	3.78 (0.80)	3.35 (0.62)		
Community activity participation	Rarely	3.11 (0.73)	3.36 (0.86)	3.14 (0.51)	20.46	<0.001
	Sometimes	3.10 (0.87)	3.53 (0.73)	3.25 (0.59)		
	Often	3.38 (1.17)	3.95 (0.72)	3.46 (0.70)		
Willingness to seek help	None	2.77 (0.84)	3.56 (0.79)	3.15 (0.64)	39.27	<0.001
	Low	2.95 (0.63)	3.33 (0.72)	3.13 (0.45)		
	High	3.59 (0.94)	3.75 (0.82)	3.39 (0.65)		

### 3.4 Multivariate analysis of social integration and satisfaction with primary care

We conducted a multivariate analysis with satisfaction with primary care as the dependent variable. Model 1 included three variables representing social integration, Model 2 included economic district variables, and Model 3 incorporated control variables such as culture and income. The results demonstrated the robustness of the models.

In Model 3, after controlling for relevant variables, community activity participation, district economic development level, neighborhood interactions, willingness to seek help, average monthly income, gender, and marital status all had statistically significant effects on satisfaction with primary care (*p* < 0.05). Specifically, compared to non-participants, those who occasionally and frequently participated in community activities reported increased satisfaction scores by 0.08 (*p* = 0.016) and 0.28 (*p* < 0.001) points, respectively.

Frequent neighborhood interactions increased satisfaction by 0.11 (*p* = 0.048) points compared with those with no interactions. The willingness to seek assistance from street offices or resident committees when faced with difficulties increased satisfaction by 0.37 (*p* < 0.001) points compared to those unwilling to seek help. In terms of regional economic influence, satisfaction scores increased by 0.37 (*p* < 0.001) and 0.10 (*p* = 0.020) points in areas with moderate and good economic conditions, respectively, compared with less economically developed areas. Females reported lower satisfaction than males. Married individuals had higher satisfaction, with an increase of 0.08 (*p* = 0.043) points compared to unmarried or divorced individuals. For those with a monthly income between 3,501 and 6,500 yuan, satisfaction decreased by 0.09 (*p* = 0.015) points compared to those earning 3,500 yuan or less (Table 4).

**Table 4:** Multivariate analysis of social integration and primary care satisfaction.

Variable		Model 1	Model 2	Model 3
		β (95% CI)	β (95% CI)	β (95% CI)
Neighborhood interactions	Rarely	Ref	Ref	Ref
	Sometimes	−0.03 (−0.11, 0.06)	−0.07 (−0.15, 0.01)	−0.06 (−0.14, 0.03)
	Often	0.15 (0.04, 0.25)^**^	0.09 (−0.01, 0.20)	0.11 (0, 0.21)^*^
Community activity participation	Rarely	Ref	Ref	Ref
	Sometimes	0.08 (0.01, 0.15)^*^	0.09 (0.02, 0.16)^*^	0.08 (0.02, 0.15)^*^
	Often	0.32 (0.21, 0.43)^***^	0.29 (0.19, 0.40)^***^	0.28 (0.18, 0.39)
Willingness to seek help	None	Ref	Ref	Ref
	Low	−0.03 (−0.12, 0.05)	−0.01 (−0.09, 0.07)	−0.02 (−0.10, 0.07)
	High	0.35 (0.26, 0.44)^***^	0.37 (0.29, 0.46)^***^	0.37 (0.28, 0.46)^***^
Levels of GDP	Low		Ref	Ref
	Medium		0.40 (0.32, 0.48)^***^	0.37 (0.29, 0.46)^***^
	High		0.12 (0.04, 0.19)^**^	0.10 (0.02, 0.18)^*^
Gender	Male			Ref
	Female			−0.07 (−0.13, 0)^*^
Age	≤29			Ref
	30–39			0.01 (−0.08, 0.11)
	≥40			−0.08 (−0.19, 0.03)
Education	Primary and lower			Ref
	Junior/senior high			−0.14 (−0.30, 0.02)
	College and above			−0.06 (−0.23, 0.11)
Marriage	Single/divorced			Ref
	Married			0.08 (0, 0.16)^*^
Hukou registration	Rural			Ref
	Urban			−0.03 (−0.10, 0.04)
Monthly income (RMB)	≤3,500			Ref
	3,501–6,500			−0.09 (−0.16, -0.02)^*^
	≥6,500			0.01 (−0.10, 0.12)
Duration of residence	≤ 1 year			Ref
	1–5 years			0.06 (−0.02, 0.13)
	≥ 5 years			0.08 (−0.02, 0.18)
Occupation	Other			Ref
	Manufacturing Industry			−0.01 (−0.12, 0.11)
	Service Industry			−0.02 (−0.10, 0.06)

*p < 0.05, **p < 0.01, ***p < 0.001.

## 4 Discussion

Our study revealed moderate satisfaction with primary care, but insufficient community integration among migrant populations in Guangzhou. Meanwhile, both community integration and district-level economic development significantly influenced migrants’ satisfaction with primary care.

Our study reported the migrant population’s satisfaction with primary care score of 3·29 ± 0·76 out of 5, indicating a moderate level. This finding aligns with other research in the Greater Bay Area. For instance, Huo assessed the primary care experiences of migrants in the Guangdong-Hong Kong-Macao Greater Bay Area using the Primary Care Assessment Tools (PCAT), reporting an average score of 3.12 ± 0·02 out of 4. Among subdomains, scores for first-contact care in terms of access and ongoing care were lowest, at 2.89 and 2.98 respectively, reflecting migrants’ limited recognition of core primary care attributes such as accessibility and continuity of care (29). International migration studies similarly identify access to care as a major barrier, contributing to migrants’ lower primary care satisfaction compared to native populations (30–32). This study also found that positive social integration significantly influences migrants’ satisfaction with primary healthcare, suggesting that enhancing social integration may help overcome access barriers and improve satisfaction with primary care.

We also found that community integration among migrants in Guangzhou is generally low, with over 80% reporting little or no participation in community activities. This low engagement poses a significant barrier to both community integration and access to primary care. Several factors may contribute to this phenomenon. Occupational segregation may diminish migrants’ sense of identity, as their social networks often remain confined within their professional circles, limiting interaction with local residents and reducing involvement in community activities (33). Long working hours and demanding jobs leave migrants with insufficient time and energy to participate. Third, community activities tend to cater predominantly to local residents’ interests-such as older adult dance groups or cultural lectures-while neglecting migrants’ needs, including vocational training or cross-regional social events (34). Moreover, activities are frequently scheduled during weekday daytime hours, conflicting with migrants’ work schedules. The information dissemination is often inefficient; reliance on traditional methods like community bulletin boards, combined with migrants’ limited access to local communication channels, restricts timely awareness of available activities (35). Addressing these barriers is essential to enhance migrants’ community participation and, consequently, their social integration and access to primary care.

Migrants in Guangzhou who participate in community activities report higher satisfaction with community health services, consistent with the findings of Feng et al. (36). Participation in community activities provides migrants with direct exposure to community health services and more comprehensive, tangible information about these services. In China, coordinated efforts among primary healthcare institutions, grassroots government agencies (such as subdistrict offices and neighborhood committees), and social organizations promote health education through distributing health materials, setting up information boards, and disseminating disease prevention knowledge. Health promotion activities, including free clinics and health lectures, further facilitate migrants’ engagement with primary care, thereby enhancing their awareness and satisfaction (37).

Our study found that migrants with more frequent neighborhood interactions reported higher satisfaction with primary healthcare, consistent with the findings of Kong et al. (38). The reasons for limited neighborhood interaction in Guangzhou are multifaceted. The significant spatial separation between residential and work areas results in low overlap in daily life trajectories, hindering the formation of intertwined social networks and depriving migrants of natural opportunities and social ties to engage in community activities (35). Many low-income migrants cluster in urban villages or peripheral communities, creating physical segregation from local residents and limiting cross-network interactions (39). Third, unique linguistic and cultural barriers in Guangdong-such as the Cantonese dialect-impede migrants’ integration into local social settings, while differences in traditional festivals may further contribute to cultural alienation (40). These factors underscore the need for grassroots governance in Guangzhou to actively create opportunities for neighborhood interaction, as increased resident engagement fosters mutual understanding and trust, thereby enhancing utilization and satisfaction with community health services (36, 41).

It is also found that fewer than one-third of migrants expressed a strong willingness to seek help from grassroots government agencies (including subdistrict offices or neighborhood committees) when facing difficulties. This low help-seeking intention may stem from migrants’ limited familiarity with urban community participation and assistance mechanisms, placing them at a disadvantage in accessing community service resources and leading them to either forgo help or seek alternative channels (42, 43). Meanwhile, data indicate that a higher willingness to seek help from grassroots government agencies significantly improves migrants’ satisfaction with primary care services. This may be because migrants who are willing to seek help have established stronger social ties and trust with local government, thereby enhancing their recognition of primary care (44). Migrants’ social networks are typically confined to their residential communities, and their willingness to seek help reflects a high dependence on community governance and service institutions, underscoring the critical role of grassroots government in delivering essential public health products and services (45). Therefore, it is recommended that grassroots government address the current lack of dedicated public welfare or volunteer groups serving migrants, which limits the provision of public services and support (24).

The migrants living in economically better-off districts reported higher satisfaction with primary care compared to those in less developed regions. This finding confirms our hypothesis that districts with better economic development can offer more fiscal directions and allocate more resources to healthcare services and community activities, whereas economically disadvantaged areas may face constraints due to limited fiscal revenue (46). Similar findings have been reported internationally, where migrants in high-income countries tend to express higher satisfaction with public health services (47). Interestingly, we also observed that migrants in districts with better economic level exhibited lower levels of neighborhood interaction, community participation, and willingness to seek assistance from grassroots government. This suggests an inverse relationship between district economic development and migrants’ willingness to integrate into community integration in Guangzhou. Therefore, it is advisable for local authorities in Guangzhou to integrate community integration initiatives with primary care and other public services, promoting coordinated implementation (48).

Furthermore, our findings indicate that female migrants and those with middle-level income reported significantly lower satisfaction with primary care. This underscores the need for policymakers to pay closer attention to the specific service needs and quality preferences of these subgroups and to provide targeted support and interventions accordingly (49, 50).

## 5 Conclusion

This study investigated the satisfaction of migrant populations in Guangzhou with primary healthcare and examined the relationships between social integration, regional economic development, and community health services. The findings indicate that migrants’ satisfaction with community health services is moderate. Both community integration and the level of district economic development positively influence migrants’ satisfaction with primary care. Therefore, we recommend that all levels of government in Guangzhou not only strengthen the infrastructure of public services but also focus on the channels and programs for enhancing community integration. Encouraging migrants to engage in public activities through community platforms can effectively promote the utilization of community health services.

## 6 Limitations

First, due to constraints in time and funding, this study collected data solely from Guangzhou, limiting regional representativeness. Future research should expand the geographic scope to enhance generalizability. Second, as this study employed a cross-sectional design, causal relationships cannot be established. Third, objective health indicators such as physical examination results and blood test data were not included due to data collection limitations. Future studies should consider the role of disease severity and overall health status in mediating the relationship between social integration and satisfaction with primary healthcare. Fourth, it is important to note that treatment burden is a critical factor affecting patients’ access to primary care; however, this study did not address this aspect. Subsequent research should comprehensively incorporate treatment burden alongside other factors to better elucidate the multifaceted determinants of primary care accessibility and utilization, thereby providing a more complete understanding of the issue.

## Data Availability

The raw data supporting the conclusions of this article will be made available by the authors, without undue reservation.
