# Supplementary material for: Community integration enhances migrants’ satisfaction with primary care across districts with varying economic levels: survey evidence from Guangzhou, China
Source: Front Public Health. 2025 Jun 11;13:1604736. doi: 10.3389/fpubh.2025.1604736 (PMC12187602; doi:10.3389/fpubh.2025.1604736)
Supplement: Supplementary file 1 [file Table_1.docx]

Since publicly available data on the migrant population could not be obtained, in this study, the migrant population was defined as the difference between the resident population and the household population, serving as a proxy for the sampling frame in our estimation procedures. Using data from the 2018 year-end statistics for each district in Guangzhou (sourced from the Guangzhou Statistical Yearbook 2019), the migrant population in 7 central districts accounted for 83.98% of the city’s total migrant population (472.57/562.75 × 100%).

**Appendix table 1.** Household population, resident population and migrant population in each district of Guangzhou

| Districts | Household population | Resident population | Migrant population | Total migrant population/percentage |
| --- | --- | --- | --- | --- |
| Liwan District | 74.54 | 97.00 | 22.46 |  |
| Yuexiu District | 117.79 | 117.89 | 0.10 |  |
| Haizhu District | 105.59 | 169.36 | 63.77 |  |
| Tianhe District | 93.92 | 174.66 | 80.74 |  |
| Baiyun District | 103.34 | 271.43 | 168.09 |  |
| Huangpu District | 52.76 | 111.41 | 58.65 |  |
| Panyu District | 98.94 | 177.70 | 78.76 | 472.57 |
| Huadu District | 78.24 | 109.26 | 31.02 |  |
| Nansha District | 43.93 | 75.17 | 31.24 |  |
| Conghua District | 63.49 | 64.71 | 1.22 |  |
| Zengcheng District | 95.15 | 121.85 | 26.70 |  |
| Total | 927.69 | 1490.44 | 562.75 | 83.98% |
